# Nanogel-Facilitated In-Situ Delivery of a Cataract Inhibitor

**DOI:** 10.3390/biom11081150

**Published:** 2021-08-04

**Authors:** Dixa Gautam, Michelle G. Pedler, Devatha P. Nair, Jonathan Mark Petrash

**Affiliations:** 1 Department of Craniofacial Biology, School of Dental Medicine, University of Colorado|Anschutz Medical Campus, Aurora, CO 80045, USA; Dixa.Gautam@CUAnschutz.edu (D.G.); Devatha.Nair@CUAnschutz.edu (D.P.N.); 2 Department of Ophthalmology, University of Colorado School of Medicine|Anschutz Medical Campus, Aurora, CO 80045, USA; michelle.pedler@CUAnschutz.edu; 3 Department of Pharmaceutical Sciences, Skaggs School of Pharmacy and Pharmaceutical Sciences, University of Colorado|Anschutz Medical Campus, Aurora, CO 80045, USA

**Keywords:** posterior capsule opacification, cataract, nanogel, drug delivery, aldose reductase, Sorbinil

## Abstract

Cataracts are a leading cause of blindness worldwide. Surgical removal of cataracts is a safe and effective procedure to restore vision. However, a large number of patients later develop vision loss due to regrowth of lens cells and subsequent degradation of the visual axis leading to visual disability. This postsurgical complication, known as posterior capsular opacification (PCO), occurs in up to 30% of cataract patients and has no clinically proven pharmacological means of prevention. Despite the availability of many compounds capable of preventing early steps in PCO development, there is currently no effective means to deliver such therapies into the eye for a suitable duration. To model a solution to this unmet medical need, we fabricated acrylic substrates as intraocular lens (IOL) mimics scaled to place into the capsular bag of the mouse lens following a mock-cataract surgery. Substrates were coated with a hydrophilic crosslinked acrylate nanogel designed to elute Sorbinil, an aldose reductase inhibitor previously shown to suppress PCO. Insertion of the Sorbinil-eluting device into the lens capsule at the time of cataract surgery resulted in substantial prevention of cellular changes associated with PCO development. This model demonstrates that a cataract inhibitor can be delivered into the postsurgical lens capsule at therapeutic levels.

## 1. Introduction

Posterior capsule opacification (PCO) is a condition that develops in as many as 50% of cases following surgery for senile cataract, the most common cause of lens opacification [1]. PCO develops from a process of epithelial-to-mesenchymal transition (EMT) of lens epithelial cells left behind after surgical removal of the cataractous lens, either through phacoemulsification or bulk removal of the lens mass mediated by hydrodissection. After going through EMT, lens epithelial cells deposit extracellular matrix components that cause contraction, wrinkling of the lens capsule, and interruption of the light path that would otherwise focus on the retina to stimulate photoreceptors. Restoration of a clear light path can be achieved by creating a capsulotomy with use of a Nd:YAG laser. While the YAG procedure is usually successful and uneventful, eyes with such capsulotomies are at higher risk for retinal detachment, cystoid macular edema, and increased ocular pressures [2]. Therefore, academic and industry teams are actively working to develop therapeutic agents to reduce the need for YAG therapy.

Many studies have shown that growth factors such as TGF-β play an important role in regulating the development of PCO [3]. Studies have demonstrated in cell culture and preclinical animal models that inhibitors of TGF-β signaling can suppress EMT and changes in lens epithelial cells involved in PCO [4,5,6]. In the ideal case, such inhibitors would be deposited into the lens capsular bag at the time of surgery and inhibit TGF-β signaling either acutely or in an extended time-dependent fashion through slow release from a drug depot fabricated from nanogel materials.

In the current study, we created a nanogel-based drug delivery matrix to slowly release a small molecule PCO inhibitor into the capsular bag in a mouse cataract model. Profiling of the post-surgical mouse eye showed that the drug delivery device was capable of delivering amounts of the PCO inhibitor sufficient to suppress markers of PCO over a 5-day period. These results suggest that nanogel-facilitated delivery of therapeutic agents into the postsurgical lens capsule may provide an effective means to delay or prevent development of PCO.

## 2. Materials and Methods

### 2.1. Materials

2-Hydroxethyl acrylate (HEA) 96%, 2,2′-azobis(2-methylpropionitrile) (AIBN) 98%, and 2-hydroxyethyl methacrylate (HEMA) were all obtained from Sigma-Aldrich (St. Louis, MO, USA). Tetraethylene glycol dimethacrylate (TTEGDMA-pure grade with MEHQ) and urethane dimethacrylate (UDMA) were both purchased from Esstech Inc. (Essington, PA, USA), whereas acrylic acid (AA) > 99.0% was purchased from Tokyo Chemical Industry Co., Ltd. (Tokyo, Japan). 2-mercaptoethanol (ME) 99% was purchased from Acros Organics (Fair Lawn, NJ, USA). Ebecryl 270™ (EB270) and Genocure* LTM were kindly donated by Allnex (Alpharetta, GA, USA) and RAHN USA Corp. (Aurora, IL, USA), respectively, and all solvents used were obtained from Fisher.

### 2.2. Synthesis of Nanogel

A previously published synthetic protocol was adapted for synthesis of the nanogel [7]. Briefly, a 60:20:20 molar ratio of HEA:TTEGDMA:AA using 15 mol % of ME as a chain transfer agent was initially synthesized herein denoted as HTA NG. Next, 1 wt.% of the thermal initiator 2,2′-azobis(2-methylpropionitrile) (AIBN) was added and stirred at 200 RPM (80 °C) using 4× methyl ethyl ketone (MEK) as the solvent and stopped at 70% conversion. The double bond conversion of the acrylate groups was monitored via FTIR spectroscopy (mid-IR, 814 cm^−1^). Once the 70% double-bond conversion was achieved the nanogel was terminated by precipitating in 10-fold excess hexanes. Subsequently, the nanogel was dispersed in dichloromethane and a stoichiometric quantity of ME was added to the nanogels such that 20% of the remaining double-bonds could undergo a thiol-Michael reaction. The reaction would provide the nanogels with OH functionality on the surface, thereby increasing their hydrophilicity while still retaining residual acrylates which could enable the nanogels to covalently link onto a substrate. Any residual solvent was then removed completely via a rotary evaporator until a gel-like NG was achieved.

The molecular weight and size of the NG was analyzed via the Viscoteck-270 gel permeation chromatograph (GPC) with tetrahydrofuran (THF) (0.35 μL/min) as the mobile phase.

### 2.3. Eb270/HEA Substrate Fabrication

For benchtop experiments, circular substrates of 6 mm in diameter and 1 mm in thickness were fabricated by casting a 2:1 weight ratio of Eb270:HEA into silicon molds and photopolymerizing them at room temperature. The samples were clamped between two glass slides that were cured with a broad spectra lamp (365–600 nm, 36 W/cm^2^) using 1 wt.% Genocure* LTM as the photo initiator. Substrates used for in vivo testing were fabricated with dimensions of 1 mm in diameter by 0.5 mm in thickness using the same method described above.

The modulus of elasticity of the IOL before and after the coating was placed was characterized on a Materials Testing System (MiniBionix II, MTS, Eden Prairie, MN, USA) by generating stress-strain graphs. Bar specimens (25 mm × 2 mm × 2 mm, *n* = 5) of the polymerized IOL with and without the coating were subject to flexural loading using a 100 N load cell at a rate of 1 mm/min and the modulus of elasticity was calculated.

### 2.4. Encapsulating Sorbinil in Nanogels

A 2 mg/mL Sorbinil solution was prepared by sonicating 10 mg of Sorbinil with 0.75 mL of 0.1 M NaOH until dissolved completely. Next, 4 mL of phosphate-buffered saline was added to solution and then separated into 1 mL aliquots. When ready to use, samples were thawed via sonication and 10 uL of 0.1 M HCL was added to the 1 mL aliquot.

A 1:2 w/v of the nanogel was dispersed in the Sorbinil solution described above and incubated overnight at ambient temperature. The mixture was centrifuged with a centrifugal concentrator (MWCO 10 kDa, polyether sulfone membrane) at 4300 RPM followed by a Milli-Q wash. The filtrate was collected and diluted from this sample where the encapsulation efficiency of the nanogel was then calculated given the following equation:

$$Encapsulation\;Efficiency\;\left(\%\right)=\frac{Total\;Sorbinil\;Mass-Qunatified\;free\;Sorbinil}{Total\;Sorbinil\;Mass}\;\times \;100$$

The optical clarity of the Sorbinil-encapsulated nanogel was studied via UV-Visible spectroscopy on a 96-well plate using Biotek Synergy 4 microplate reader (BioTek Instruments, Inc., Winooski, VT, USA), in which 3 µL of the Sorbinil-encapsulated nanogel was added in each well plate well (*n* = 3) to confirm Sorbinil was encapsulated and no absorbace was detected in the visible region (400–700 nm). 

### 2.5. Drug Elution from NG Devices

To measure Sorbinil release from the HTA NG, 3 µL of the Sorbinil-loaded NG was spin coated on top of the benchtop substrates then cured with a broad spectra lamp (365–600 nm, 36 W/cm^2^) using 0.1 wt.% Genocure *LTM as the photo initiator. To monitor drug release, coated substrates were submerged in a 10 mL 1×-PBS reservoir at 24 °C. For each reading, 1 mL samples were collected from the reservoir at 30 min, 1 h, 3 h, 7 h, and 1, 2, 3, 5, and 7 days. This benchtop study was designed to baseline the maximum amount of Sorbinil that could be removed from the nanogels into the reservoir via diffusion, therefore the reservoir (10 mL) was replaced at the 7 h mark and subsequently at days 1, 2, 3, 5, and 7. Additionally, the reservoir was stirred at 100 RPM to expedite the removal of Sorbinil from the nanogels. Sorbinil was detected by measuring absorbance of 280 nm light. The readings were then normalized relative to the theoretical maximum concentration of Sorbinil released given by that encapsulation efficiency calculated earlier.

### 2.6. Sterile Sample Preparation

Substrates were sterilized in an autoclave (Tuttnauer, 2540 M-B/L Heiddolph, Alexander, AK, USA) at 265 °F for one hour followed by a dry cycle at the same temperature and duration. Then, 1 µL of the Sorbinil loaded NGs, filter sterilized using a Celltreat 0.22 µm filter (Pepperell, MA, USA), was pipetted on top of the substrate and cured with a broad spectra lamp (365–600 nm, 36 W/cm^2^) using 0.1 wt.% Genocure *LTM as the photo initiator. Samples were prepared 24 h before surgery.

### 2.7. Mouse PCO Model

For animal studies of PCO, we utilized our previously published mouse model wherein a transgene encoding human aldose reductase (AKR1B1) accelerates the onset and progression of PCO following lens extraction [6,8]. Animals were anesthetized using 90 mg/kg ketamine (VetOne, Cambridge, ON, Canada) and 10 mg/Kg xylazine (VetOne, Cambridge, ON, Canada), and given 1 mg/kg buprenorphine SR (ZooPharm, Laramie, WY, USA). The pupils were then dilated with one eye drop of 0.2% tropicamide (Akron, Lake Forest, IL, USA), 0.5% phenylephrine (Akron, Lake Forest, IL, USA), and the cornea anesthetized with one drop of ophthalmic proparacaine (Alcon, Geneva, Switzerland) and 5% ophthalmic betadine (Alcon, Geneva, Switzerland). Extra capsular lens extraction (ECLE) was performed by making an incision through the cornea and the lens capsule using a 1 mm slit scalpel (Alcon, Geneva, Switzerland) followed by hydrodissection of the lens fiber mass using saline in a 5 mL syringe with a bent cannula (Alcon, Geneva, Switzerland). For animals that received EB270:HEA substrates, nanogel loaded with or without Sorbinil combinations was placed through the anterior chamber into the lens capsule. The anterior chamber volume was restored using viscoat (Alcon, Geneva, Switzerland) if needed and the cornea opening was sealed using Resure™ (Ocular Therapeutix, Bedford, MA, USA). A drop of dexamethasone, polymyxin B, and neomycin ophthalmic gel (Bausch and Lomb, Bridgewater, NJ, USA) was placed over the corneal incision. The animals recovered for 5 days before imaging, then the lens capsule was recovered for RNA analysis or the whole eye fixed for immunohistochemistry studies.

### 2.8. Histology

Whole eyes were fixed in 4% paraformaldehyde for 10 min at 4 °C followed by 4 h per each sucrose (Sigma, St. Louis, MO, USA) gradient 10%, 20%, 30%, and then frozen into optimal cutting temperature (OCT, Tissue Tek, Torrance, CA, USA). The sections were stained with 1:500-diluted αSMA A488 antiserum (Abcam, ab202295, Cambridge, MA, USA) in 1% BSA (Sigma, St. Louis, MO, USA) and 0.1% Tween 20 (Fisher, Waltham, MA, USA) in saline for 1 h at room temperature. The slides were then washed and counter-stained with fluoromount (Fisher, Waltham, MA, USA) containing DAPI. They were imaged on a Nikon Eclipse Ti confocal microscope (Nikon, Tokyo, Japan).

### 2.9. qRT-PCR

RNA was extracted from the lens capsule which was placed into 500 µL Qiazol (Qiagen, Austin, TX, USA):100 µL chloroform which was immediately frozen to −80 °C following extraction using RNeasy micro kit (Qiagen, Austin, TX, USA) according to the manufacturer’s instructions. Complimentary DNA was created using iScript reverse transcription supermix for qRT-PCR (Biorad, Hercules, CA, USA) and the qPCR was performed with iTaq Universal SYBR green supermix (Biorad, Hercules, CA, USA). We used four sets of primers (Integrated DNA Technologies, Coralville, IA, USA) for gene expression markers of EMT αSMA, fibronectin, vimentin, and E-cadherin, and GAPDH was used as the internal ubiquitous control. The primer sequences are listed in Table 1. Reactions were performed in triplicate using the CFX (Biorad, Hercules, CA, USA).

### 2.10. Statistical Analysis

Statistical analysis was performed using GraphPad Prism software 5.03 (GraphPad Software, La Jolla, CA, USA). Power calculations to estimate sample sizes to achieve 95% confidence intervals carried a maximum error of 4.615.

## 3. Results

### 3.1. Characterization of HTA Nanogel and Eb270/HEA Substrates

The HTA nanogel synthesis followed a previously published procedure and was monitored by tracking the double bond conversion of the acrylate groups until 70% conversion was achieved. Residual 10% acrylate functionality was confirmed via mid-FTIR after the addition of ME. The molecular weight and hydrodynamic radius of the HTA NG were measured to be 57,000 Da and 9.3 nm, respectively. Encapsulation efficiency was calculated to determine the Sorbinil encapsulated by the nanogel. A standard curve of Sorbinil ranging from 0–10 µg/mL was prepared to relate absorbance to concentration on a 96-well microplate (λ_abs_ = 280 nm). After the Sorbinil was loaded into the nanogel, washed, and centrifuged, the free Sorbinil was collected, and the encapsulation coefficient was determined to be 83 ± 5%. For in vivo substrates, the amount of Sorbinil encapsulated was calculated to be 0.33 ± 0.06 µg.

**Figure 1:**
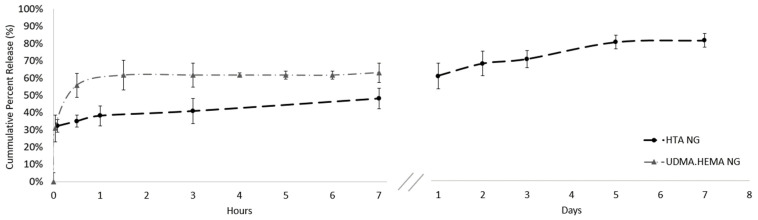
Sorbinil release in phosphate buffered saline from HTA NG over a period of 7 days compared to the burst release of Sorbinil from the UDMA:HEMA NG over a 7 h period. These drug release averages *n* = 3 experimental replicates as well as *N* = 3 technical replicates.

Several other formulations were fabricated before finalizing the use of the HTA nanogel. These formulations utilized various ratios of hydrophobic/hydrophilic monomers such as urethane dimethacylate (UDMA) and 2-hydroxyethyl methacrylate (HEMA). These nanogel systems (40:60 UDMA:HEMA NG) however, only had encapsulation efficiencies of approximately 30% and almost all Sorbinil was released within two hours. The covalent addition of the 2-mercaptoethanol on the outer surface of the HTA nanogel allowed for pendent OH groups which in turn, led to more diffusion of the Sorbinil within the nanogel yielding a higher encapsulation efficiency [9]. The Sorbinil-loaded nanogels were coated on substrates, and photopolymerized in place with a broad spectra lamp (365–600 nm, 36 W/cm^2^) again for 5 min. To baseline the maximum amount of Sorbinil eluting that could be removed from the nanogels into the reservoir via diffusion, the reservoir (10 mL) was replaced at the 7 h mark and subsequently at days 1, 2, 3, 5, and 7. While it is noted that the flow of aqueous humor can affect the elution rate of the drug in vivo and that simple drug diffusion varies as a function of temperature, the initial tests conducted under set conditions helped us baseline the release of the nanogels for this study. The residual acrylate groups on the nanogel covalently crosslink, both onto the outer surface of the nanogel and with each other, thereby creating a crosslinked mesh work that can curb the burst release and deliver [7,9] the sustained release of Sorbinil shown in Figure 1.

To further characterize nanogel coated and uncoated substrates, elastic modulus and swelling were tested. As shown in Figure S1, the tested bar specimens showed no significant difference in elastic modulus between coated (8.89 ± 0.52 MPa) and uncoated samples (9.07 ± 0.46 MPa). The swelling of coated and uncoated substrates in distilled water indicated that there is no significant difference between both coated and uncoated sample types (Figure S2). The weights of the substrates (*n* = 3) were taken before being placed in 5 mL of distilled water at 37 °C, then weighed again at both the 24 and 48 h time points. As the substrates have high double-bond conversions ((meth) acrylate conversion of 99.20 ± 0.37), only 9.3 ± 2.3% and 10.2 ± 1.8% was observed for uncoated and coated substrates, respectively. The HTA nanogel showed negligible absorbance of light across visible and ultraviolet wavelengths. As expected, HTA nanogel with encapsulated Sorbinil showed an absorbance peak at approximately 280 nm corresponding to Sorbinil (Figure S3).

### 3.2. Post-Surgical Tolerability of Drug Delivery Device in the Mouse Eye

**Figure 2:**
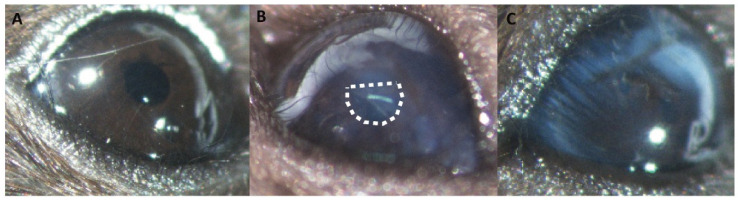
Tolerability of the EB270/HEA drug delivery substrate in the mouse eye. Eyes were examined 5 days following surgical removal of the native lens mass. In comparison with the unoperated eye (panel **A**), eyes with the native lens replaced with the EB270/HEA substrate (panel **B**) or following lens extraction alone (panel **C**) showed no obvious signs of inflammation or irritation. The intraocular location of the substrate is outlined by a dashed line in panel (**B**).

Since we were unaware of published work describing deployment of an intraocular device fabricated from EB270/HEA, we first tested the tolerability of our substrates placed into the lens capsule following removal of the native lens mass. As shown in Figure 2, no obvious signs of inflammation or irritation were observed in the mouse eye 5 days after placement of an EB270/HEA substrate.

### 3.3. Efficacy of Anti-Cataract Drug Delivered via Nanogel-Based Device

**Figure 3:**
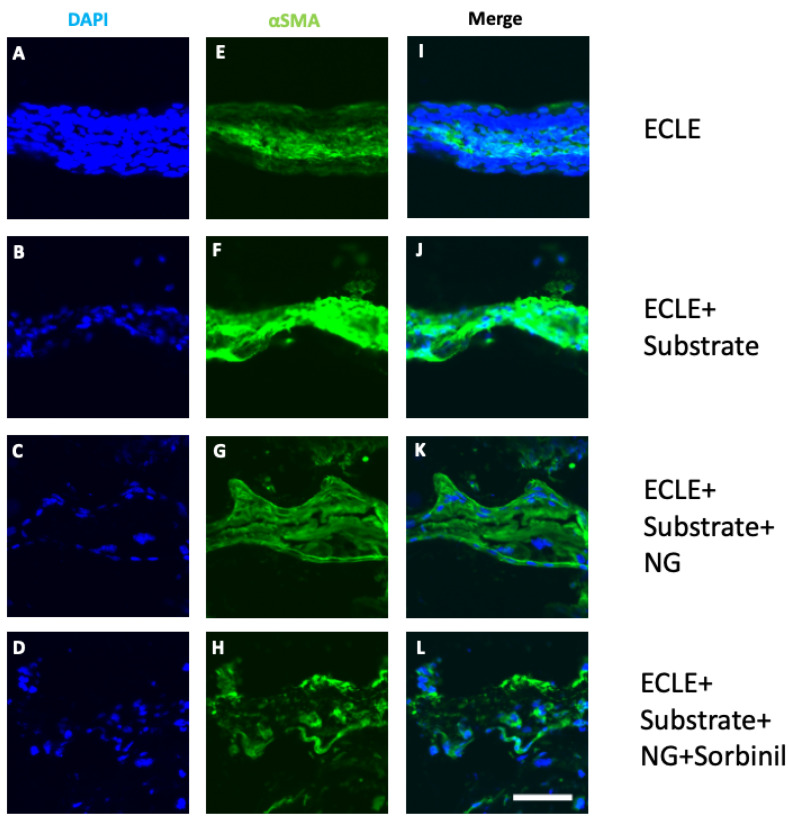
Postsurgical immunofluorescence staining for αSMA in mouse lens capsule. Capsular bags were recovered 5 days following surgery. Treatment groups included surgery only (ECLE, panels **A**,**E**,**I**), ECLE with naked substrate (panels **B**,**F**,**J**), ECLE with nanogel-coated substrate (panels **C**,**G**,**K**), and ECLE with substrated coated with Sorbinil-eluting nanogel (panels **D**,**H**,**L**). Images are representative of three separate experiments. Signals were generated after staining for nuclei with DAPI (panels **A**–**D**) and for αSMA (panels **E**–**H**). Panels (**I**–**L**) show merged signals. Scale bar is 50 μm.

To assess the efficacy of Sorbinil released via our nanogel-based materials, we immunostained for αSMA, a marker for EMT associated with early stages of PCO. In all cases, we studied lens capsular bags harvested from the eyes of mice 5 days after lens extraction surgery. Immunostaining intensity, which is semiquantitative for the abundance of αSMA in this case, provides a reasonable indication for the abundance of this marker. We included three therapeutic control conditions for our study, each comprising postsurgical capsular bags treated with increasingly complex therapeutic components. These controls included capsular bags with no external components inserted and capsular bags containing a naked, uncoated substrate, as well as capsular bags treated with a nanogel-coated substrate. These controls were compared with capsular bags treated with substrates coated with Sorbinil-loaded nanogel. As shown in Figure 3, a strong immunostaining signal was observed in capsular bags containing the naked substrate, with progressively less intense staining observed in capsules treated with nanogel-coated substrates and even less staining with substrates coated with Sorbinil-loaded nanogel. In the latter case, reduced staining for αSMA in animals treated with nanogel-facilitated delivery of Sorbinil was similar to our previous studies involving systemic treatment with Sorbinil via drinking water [6].

**Figure 4:**
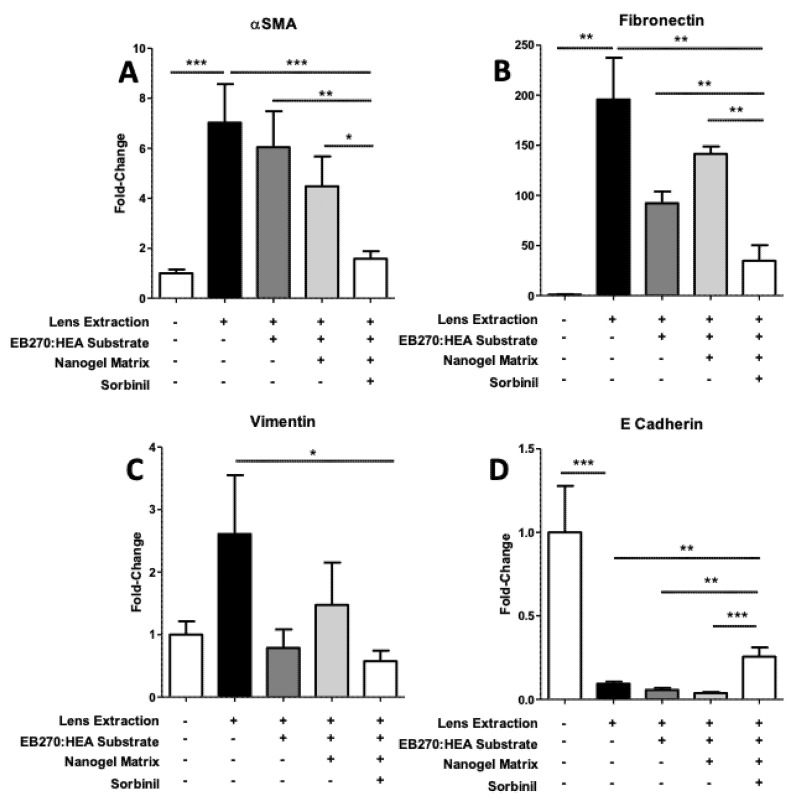
Influence of cataract inhibitor delivery system components on expression of EMT marker genes in lens capsule-epithelium. Marker genes included αSMA (**A**), fibronectin (**B**), vimentin (**C**) and E-cadherin (**D**). Presence of components arrayed beneath each reading is indicated by “+”. The degree of statistical significance of differences among comparison groups is given using one-tailed Student *t*-test, where *** *p* < 0.001, ** *p* < 0.01, and * *p* < 0.0 (*n* = 3 for each set of conditions, repeated ≥3 times).

We used qRT-PCR to estimate changes in expression of genes associated with the EMT process in mice, including αSMA, fibronectin, vimentin, and E-cadherin. As with our immunostaining experiments, we included three treatment controls in our comparison groups in order to uncover possible treatment effects of each of the drug delivery components on the induction of EMT markers in our model system. As shown in Figure 4, lens extraction led to a dramatic increase in the abundance of αSMA gene transcripts in RNA extracted from lens capsular bags. Animals treated with the placement of substrates with increasingly complex components of the Sorbinil-delivery system showed progressively less but statistically insignificant changes in αSMA gene expression. However, substrates containing Sorbinil-loaded nanogel showed significantly reduced levels of αSMA gene transcripts (*p* < 0.001). A similar pattern of greatest reduction in EMT marker expression in eyes treated with substrates coated with Sorbinil-loaded nanogel was observed in experiments targeted for fibronectin gene transcripts (*p* < 0.01). We observed a partial recovery of E-cadherin gene expression in eyes treated with substrates coated with Sorbinil-encapsulated nanogel, consistent with previous experiments demonstrating that Sorbinil treatment helps to suppress the transition of lens epithelial cells toward the mesenchymal phenotype in this surgical model.

## 4. Discussion

Cataracts are responsible for almost 11 million cases of blindness and 35 million cases of visual impairment worldwide [10]. The surgical treatment of cataracts involves removal of the cloudy lens mass and usually replacement of the native lens with an artificial device called an intraocular lens (IOL). While this procedure is usually safe and uneventful, in up to 30% of cases, and even more depending on time, patients develop a secondary cataract called posterior capsule opacification (PCO), which causes visual impairment [11]. Removal of the secondary cataract and restoration of the visual light path is most often accomplished with Nd:YAG laser capsulotomy. Because YAG laser adds expense and potential complications such as secondary glaucoma, macular edema, and retinal detachment [12], there is considerable need to prevent PCO in the first place.

PCO develops as a result of epithelial-to-mesenchymal transition (EMT) of lens epithelial cells that remain adherent to the lens capsule following cataract extraction [3]. TGF-β is thought to be a key regulator of PCO through its role in signaling downstream from its cell surface receptor to SMAD proteins [13,14,15]. While strategies to prevent PCO have focused on signaling pathways as well as structural parameters of intraocular lenses, we still do not have sufficiently durable treatments to overcome the inexorable drive of lens epithelial cells to undergo EMT and cause visual impairment in the post-cataract patient [16]. A major unanswered question is whether blockade of the EMT process that underlies PCO, if limited as in the present study to the acute period immediately following cataract removal, will be sufficient to prevent PCO at later times. This question will be addressed in animal studies to be carried out in the future.

Our previous studies identified aldose reductase (AR), an aldo-keto reductase thoroughly studied as a catalyst of sorbitol production in diabetes [17], as a key player in regulating PCO [5,6,8,17]. Unlike in humans, mouse lenses contain very low levels of aldose reductase [18]. To make a mouse model more relevant to human cataract, we cloned the human gene encoding aldose reductase into mice and demonstrated that such mice are at higher risk for cataracts [19]. We hypothesize that elevation of AR using the transgenic approach resulted in a more human-like induction of EMT markers following the mock cataract procedure, and such changes represent measurable and meaningful indices for discovery of agents to prevent or delay PCO in humans.

Small, orally active AR inhibitors such as Sorbinil substantially prevent EMT of lens epithelial cells following cataract surgery in mice [6]. In addition, intraocular deposition of a protein biologic derived from the inhibitory SMAD7 also shows promise against PCO [20]. In both cases, little is known yet about the width of the therapeutic window necessary to achieve efficacy against PCO pathogenesis. Assuming lens epithelial cells may develop a fibrotic response long after exposure to the inducing effects of TGF-β [21], it seems reasonable to assume that blockade of PCO development in human patients may require delivery of therapeutic agents over an extended period of time. Our previous studies demonstrated that systemic delivery of Sorbinil suppressed expression of EMT markers associated with fibrotic PCO but did not affect the expression of proteins typical of lens fiber cells thought to be responsible for pearl-type PCO [6]. Future studies will be needed to address the impact of nanogel-delivered inhibitors on the expression of markers for both fibrotic and pearl-type PCO at longer times following lens extraction in our mouse model.

While topical drug delivery accounts for approximately 90% of aqueous ophthalmic formulations, the biggest disadvantages include limited drug concentration and the barrier function of the cornea [22,23]. For these reasons, it would be beneficial to develop an effective system that can deliver therapeutic doses of a PCO-inhibiting molecule to the lens, which occupies space within the center of the eye and is separated from the ocular surface by several tissue and aqueous compartments. Many investigators have proposed using an IOL as a substrate to coat with drug-eluting materials. However, coating the nonrefractive IOL surfaces with drug formulations could alter the inherent properties of the IOL and introduce unacceptably complex manufacturing bottlenecks. Some early IOL-eluting prototypes have shown limited drug delivery windows of approximately 12 h [24]. Other methods, such as the addition of drug releasing capsular tension rings, have proven to be problematic, as the membrane-controlled system has risk of ruptures leading to acute toxicity when large quantities of drugs possibly burst into the eye [25]. For our studies, we considered it desirable to derive a mouse eye-sized piece of clinical IOL for coating rather than the IOL-mimic substrate used in our studies. Unfortunately, it was not possible to produce IOL fragments of a consistent size and shape which would be needed for coating and implantation in the mouse eye. Our workaround was to design a mouse-sized IOL-like substrate for our in vivo studies. Briefly, circular IOL substrates of 1 mm in diameter and 0.5 mm in thickness were generated. Subsequently, nanogel-coated IOLs were obtained when 1 uL of the Sorbinil-eluting nanogel was polymerized on the surface of the IOL substrates for the in vivo mouse studies. It is important to point out that the IOL mimic formulated for this proof-of-concept study was designed to demonstrate the feasibility of this approach to deliver an inhibitor and not to design an IOL with the intended refractive power. While drug-eluting contact lenses and intraocular lenses have been gaining significance over the past decade, to the best of our knowledge, the current study is unique in focusing on drug-eluting IOLs in which the drug-delivery carrier is a sub 10 nm pre-loaded nanogel that is covalently linked to the surface of IOL. The presence of Sorbinil within nanogel-based coatings in IOLs is also novel and has not been previously studied.

In terms of flexibility and hydrophilic nature, the EB270/HEA substrate utilized in our animal studies was designed to be similar to a typical IOL on the market but scaled to be suitable for implantation in the mouse lens capsular bag (approximately 1 mm diameter). Furthermore, since the substrate was not designed with a refractive function as would be expected for a device providing long term correction of visual acuity, we did not assess whether the materials have significant ocular toxicity. The water contact angle of the EB270/HEA substrate was measured to be 59.1 ± 3.9 degrees, showing that this substrate is clearly hydrophilic. While hydrophilic IOLs and hydrophobic IOLs perform the same, hydrophobic substrates have a higher tendency to adsorb extracellular matrix proteins and inflammatory cells which may lead to other complications. For these reasons, we chose to focus on hydrophilic substrates for our model system [26]. Our nanoscale surface coating has been shown to not affect the inherent properties of the substrate and simply provide one week of sustained release. Further studies will be required to determine the duration and boundaries of the therapeutic window for PCO prevention and thus the requirements for delivery of PCO inhibitors. Ultimately, our goal is to develop a means to effect sustained release of inhibitor so as to remove the need for multiple surgeries. As the burden of cataracts will continue to increase in parallel with increases in the aged population worldwide, so will the need for effective measures to prevent vision loss due to PCO. Further studies underway will hopefully help to bridge the gap between intraocular drug delivery technology and this unmet clinical need.

## Figures and Tables

**Table 1:** PCR primer sequences.

Gene	Forward Primer	Reverse Primer
αSMA	5′-CTGTTATAGGTGGTTTCGTGGA-3′	5′-GAGCTACGAACTGCCTGAC-3′
Fibronectin	5′-TTGTTCGTAGACACTGGAGAC-3′	5′-GAGCTATCCAATTTCACCTTCAGA-3′
Vimentin	5′-TCAACATCCTGTCTGACTG-3′	5′-ATCAGCTCACCAACGACAAG-3′
E-Cadherin	5′-AGTCTCGTTTCTGTCTTCTGAG-3′	5′-GAGCTGTCTACCAAAGTGACG-3′
GAPDH	5′-AATGGTGAAGGTCGGTGTG-3′	5′-GTGGAGTCATACTGGAACATGTAG-3′

## Data Availability

Not applicable.

## Supplementary Materials

The following are available online at https://www.mdpi.com/article/10.3390/biom11081150/s1, Figure S1: Elastic Modulus of uncoated and coated IOL substrates. The modulus of elasticity of the IOL before and after the coating was placed was characterized on a Materials Testing System (MiniBionix II, MTS, Eden Prairie, MN, USA) by generating stress-strain graphs. Briefly, bar specimens (25 mm × 2 mm × 2 mm, n = 5) of the polymerized IOL with and without the coating were subject to flexural loading using a 100N load cell at a rate of 1 mm/min and the modulus of elasticity was calculated. Results showed no significant difference in elastic modulus between coated (8.89 ± 0.52 MPa) and uncoated samples (9.07 ± 0.46 MPa). Figure S2: Swelling of uncoated and coated IOL substrates. The swelling of coated and uncoated substrates in distilled water are shown below and indicates that there is no significant difference between all substrates. The weight of the substrates (n = 3 for all groups) were taken before being placed in 5 mL of distilled water at 37 °C, then weighed again at after 24 and 48 h. As the substrates have high double-bond conversions ((meth)acrylate conversion of 99.20 ± 0.37) only 9.3 ± 2.3% and 10.2 ± 1.8% was observed for uncoated and coated substrates respectively. Figure S3: Optical absorbance of encapsulated Sorbinil nanogel coating. The optical clarity of the encapsulated sorbinil nanogel coating was studied via UV-Visible spectroscopy in which 3 µL the sorbinil encapsulated nanogel was added in each well of a 96-well microplate (*n* = 3). Sorbinil has a wavelength absorbance peak from 260−280 nm which is shown below, but no absorbance is detected in the visible region (400−700 nm). This was compared to the HTA nanogel without any nanogel, showing the optical clarity of both systems.
