# Supplementary material for: Nanogel-Facilitated In-Situ Delivery of a Cataract Inhibitor
Source: Biomolecules. 2021 Aug 4;11(8):1150. doi: 10.3390/biom11081150 (PMC8391309; doi:10.3390/biom11081150)
Supplement: Supplementary file 1 [file biomolecules-11-01150-s001.zip › biomolecules-1289898-supplementary.pdf]

Supplementary Materials

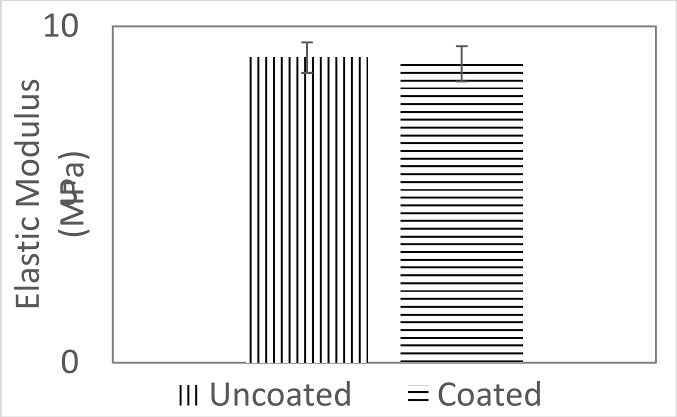

Figure S1: Elastic Modulus of uncoated and coated IOL substrates. The modulus of elasticity of the IOL before and after the coating was placed was characterized on a Materials Testing System (MiniBionix II, MTS, Eden Prairie, MN, USA) by generating stress-strain graphs. Briefly, bar specimens (25 mm x 2 mm x 2mm, n=5) of the polymerized IOL with and without the coating were subject to flexural loading using a 100N load cell at a rate of 1mm/min and the modulus of elasticity was calculated. Results showed no significant difference in elastic modulus between coated (8.89 ± 0.52 MPa) and uncoated samples (9.07± 0.46 MPa).

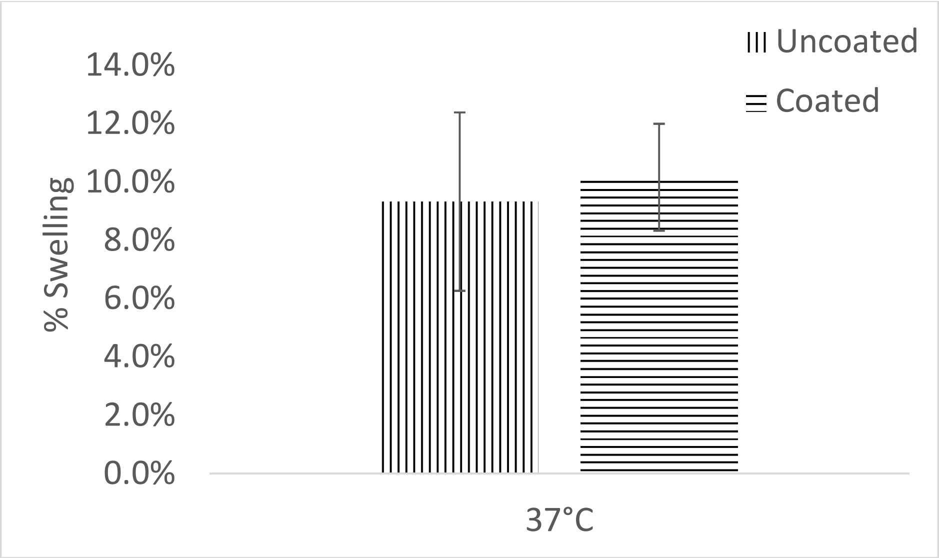

Figure S2: Swelling of uncoated and coated IOL substrates. The swelling of coated and uncoated substrates in distilled water are shown below and indicates that there is no significant difference between all substrates. The weight of the substrates (n=3 for all groups) were taken before being placed in 5mL of distilled water at 37°C, then weighed again at after 24 and 48 hours. As the substrates have high double-bond conversions ((meth)acrylate conversion of 99.20± 0.37) only 9.3 ± 2.3% and 10.2 ± 1.8% was observed for uncoated and coated substrates respectively.

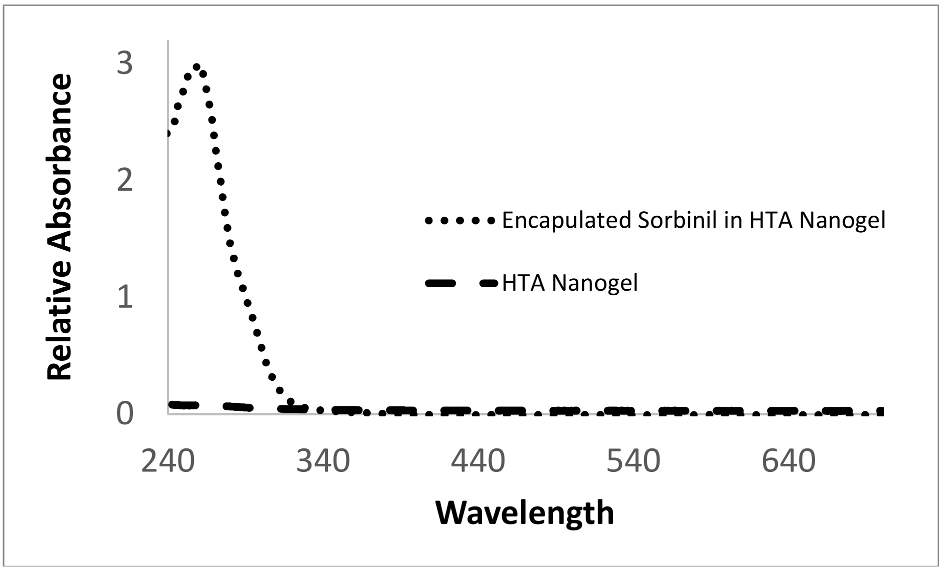

Figure S3: Optical absorbance of encapsulated Sorbinil nanogel coating. The optical clarity of the encapsulated sorbinil nanogel coating was studied via UV-Visible spectroscopy in which 3µl the sorbinil encapsulated nanogel was added in each well of a 96-well microplate (n=3). Sorbinil has a wavelength absorbance peak from 260-280nm which is shown below, but no absorbance is detected in the visible region (400nm- 700nm). This was compared to the HTA nanogel without any nanogel, showing the optical clarity of both systems.
